# Time-dependent changes in FT4 and FT3 levels measured using mass spectrometry after an acute ingestion of excess levothyroxine in a case with hypothyroidism

**DOI:** 10.1186/s13044-020-00078-7

**Published:** 2020-05-01

**Authors:** Yuko Ito, Satoru Suzuki, Yoshiko Matsumoto, Chiyo Ohkouchi, Satoshi Suzuki, Manabu Iwadate, Sanae Midorikawa, Susumu Yokoya, Shinichi Suzuki, Hiroki Shimura

**Affiliations:** 1 Department of Laboratory Medicine, School of Medicine, Fukushima Medical University, Hikarigaoka, Fukushima, Fukushima 960-1295 Japan; 2 Thyroid and Endocrinology Center, Fukushima Medical University Hospital, Fukushima, 960-1295 Japan; 3 Department of Thyroid and Endocrinology, School of Medicine, Fukushima Medical University, Fukushima, 960-1295 Japan; 4 Department of Radiation Health Management, School of Medicine, Fukushima Medical University, Fukushima, 960-1295 Japan

**Keywords:** Free thyroid hormone, Thyrotoxicosis, Congenital hypothyroidism, Excess levothyroxine, Mass spectrometry, Immunoassay

## Abstract

**Background:**

Thyrotoxicosis is common disorder among endocrine dysfunctions. It is not rare that the free thyroid hormone level exceeds the measurement range of immunoassay. Such extreme high concentration of free thyroid hormone is generally considered to be impossible to measure correctly because of changes in the balance between free hormones and binding proteins by dilution of serum. Using liquid chromatography-tandem mass spectrometry (LC-MS/MS), however, higher concentrations are able to be determined.

**Case presentation:**

We present a case of a 21-year-old female with congenital hypothyroidism who had taken a total of 5 mg levothyroxine over three consecutive days following discontinuance of the medication for a month. Immunoassay performed 3 hours after the last ingestion showed that the patient’s free thyroxine (FT4) was over 100 pmol/L and her free triiodothyronine (FT3) was 24.5 pmol/L. With a temporary cessation of levothyroxine, the patient was kept for observation without any other medication. Two days after the last ingestion, FT4 was still over 100 pmol/L and FT3 was increased to 28.8 pmol/L. After an additional 4 days, both FT4 and FT3 levels decreased. Through this period, no thyrotoxic symptom or physical sign had appeared. We also measured FT4 and FT3 levels in her cryopreserved serum by ultrafiltration LC-MS/MS. Her FT4 level measured by ultrafiltration LC-MS/MS on the visiting day and 2 days later were 160.0 and 135.5 pmol/L, respectively, indicating that the toxic dose of levothyroxine was partly changed to T3 during the 2 days. The FT3/FT4 ratios were revealed to be low, accounting for the patient’s benign clinical course despite temporal toxic exposure to levothyroxine. It is implied that prior discontinuation of supplementary levothyroxine increases potential vacant binding sites for thyroid hormone as a buffer to prevent toxic T3 effect.

**Conclusion:**

It was helpful to clarify the time dependent changes in free thyroid hormone levels by ultrafiltration LC-MS/MS in discussing the clinical course in this case. Though mass spectrometry has a disadvantage in speed for routine laboratory use, its accurate measurement, particularly of levels exceeding the measurable range of the immunoassay, provides valuable information for more appropriate management of extreme thyrotoxicosis.

## Background

Free thyroxine (FT4) and Free triiodothyronine (FT3) are commonly measured by immunoassay in the majority of clinical laboratories worldwide. Although there are some limitations to measuring thyroid hormones by immunoassay, such as influence of protein binding variations [1], autoantibodies and heterophile antibodies [2], the immunoassay method is highly sensitive, automated, and capable of handling many samples in a short turnaround time with simple pre-examination process. On the other hand, liquid chromatography-tandem mass spectrometry (LC-MS/MS), which is considered the standard method of measuring free thyroid hormone, requires specialized and very expensive equipment in addition to trained and experienced personnel. In addition, LC-MS/MS requires a longer turnaround time even if performed in-house [1]. Accordingly, until now, immunoassay might have been superior as a routine method of measurement in hospitals where rapid results are necessary, especially in emergency settings.

With regard to thyrotoxicosis, however, it is impossible to determine FT4 or FT3 concentrations by immunoassay when they are higher than the upper limit of the measurement range, because dilution of serum changes the balance between free thyroid hormone and thyroxine binding globulin. There is a report of a 3-year-old boy ingesting up to a maximum of 9 mg (0.5 mg/kg) of levothyroxine [3], and of a man who took 720 mg of veterinary levothyroxine [4]. Ingestion of such huge amounts of levothyroxine could result in tremendously high concentrations of FT4 and FT3; however, immunoassay could not measure such high FT4 levels due to its limited measurable range. In such cases, measurement using LC-MS/MS might be more practical.

In this report, we discuss the availability of thyroid hormone measurement by ultrafiltration LC-MS/MS following the presentation of a case with thyrotoxicosis whose FT4 was over the measurement range by conventional immunoassay.

## Case presentation

The patient was a 21-year-old woman with congenital hypothyroidism that had been diagnosed a month after her birth, and had been ingesting levothyroxine. She was diagnosed as having iodide organification defect, and her daily dosage of levothyroxine had been 150 μg in recent years. She had no other relevant medical history, nor relatives with thyroid disease. At a regular check at our hospital, her laboratory data revealed thyrotoxicosis but a normal thyroid stimulating hormone (TSH) level, suggesting an inappropriate secretion of TSH. She confided that she had neglected to take her medication for about a month, then ingested a total of 5 mg of levothyroxine over three consecutive days: 2 mg 2 days previous to the visiting day, 2 mg 1 day previous, and 1 mg on the visiting day, about 3 hours previous to her appointment. She did not complain of any thyrotoxic symptoms.

The patient was 150 cm tall and weighed 56 kg. Physical examination revealed that she was conscious, had a pulse of 84/min, and a blood pressure of 132/71 mmHg. Her thyroid gland was diffusely enlarged and there was no apparent finger tremor or pretibial edema.

Laboratory tests showed that the levels of FT4, FT3 and TSH were over 100 pmol/L (ref. 12–22 pmol/L), 24.5 pmol/L (ref. 3.5–6.1 pmol/L) and 2.620 mIU/L (ref. 0.50–5.00 mIU/L), respectively. Electrochemiluminescence immunoassay (ECLIA) was used for clinical measurement of FT4 (ECLusys® FT_4_ II, Roche Diagnostics GmbH, Mannheim, Germany; measurement range, 0.50–100.0 pmol/L), FT3 (ECLusys® FT_3_ III, Roche Diagnostics GmbH; measurement range, 0.40–50.0 pmol/L) and TSH (ECLusys® TSH, Roche Diagnostics GmbH). Serum thyroglobulin was 3357 ng/mL (ref. ≤33.7 ng/mL), anti-thyroglobulin antibody was 21.53 IU/mL (ref. ≤28 IU/mL), anti-thyroid peroxidase antibody was 25.58 IU/mL (ref. ≤16 IU/mL) and anti-TSH receptor antibody was < 0.300 IU/L (ref. ≤2.0 IU/L). Ultrasonography showed a multi-nodular goiter with diffuse hypervascularity.

**Fig. 1:**
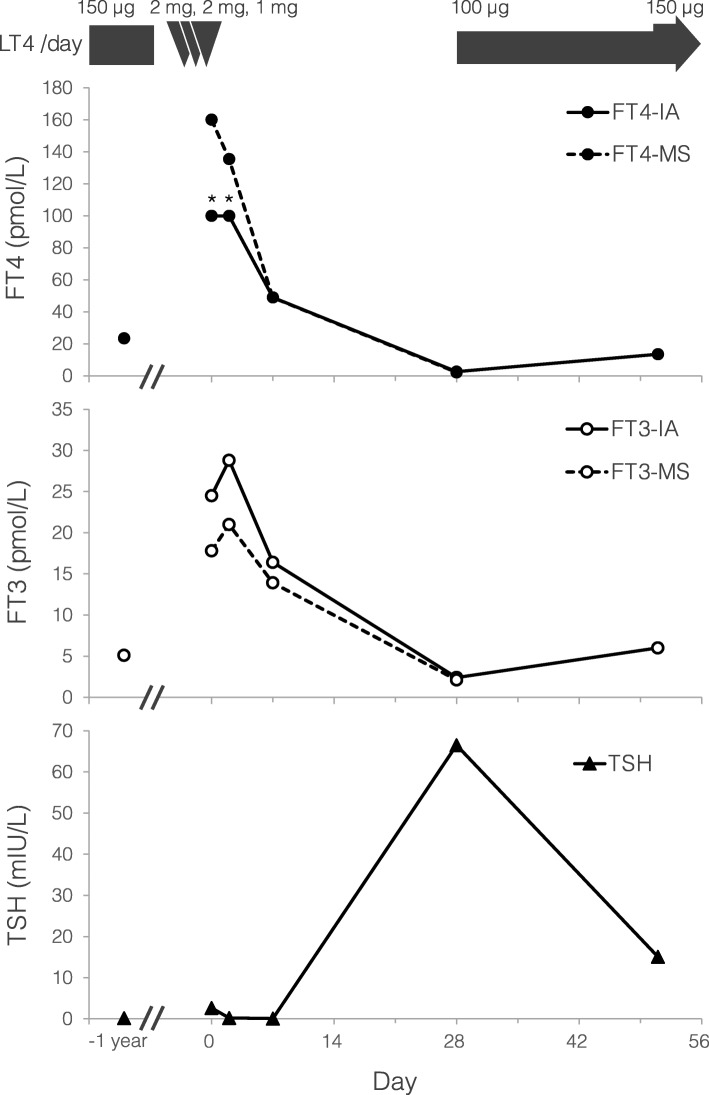
Changes in FT4, FT3 and TSH levels after the acute ingestion of 5 mg levothyroxine. The levels of FT4 and FT3 measured using ECLIA and ultrafiltration LC-MS/MS, TSH, and the daily dose levothyroxine ingested are shown. LT4, levothyroxine; IA, Electrochemiluminescence immunoassay; MS, ultrafiltration liquid chromatography-tandem mass spectrometry. * > 100 pmol/L

With a temporary cessation of levothyroxine, the patient was kept for observation without hospitalization or any other medication, because no thyrotoxic symptom or physical sign had yet appeared. Two days later, her FT4 concentration still exceeded the measurement range, and her FT3 was 28.8 pmol/L, which was higher than that on the first day. After an additional 4 days, both FT4 and FT3 levels decreased (Fig. 1, Supplementary Table 1). On the other hand, the patient’s TSH level continued to decline for 7 days. Four weeks after the first visit, these hormone levels indicated primary hypothyroidism, and she therefore resumed ingestion of levothyroxine. Through this period, she did not complain of any symptoms. Her thyroid function tests finally stayed within normal range thereafter, with 150 μg/day of levothyroxine.

For the purpose of determining the FT4 levels above the measurable range of the immunoassay, the ultrafiltration LC-MS/MS (ASKA Pharmamedical Co., Ltd., Kawasaki, Japan) was employed for quantification of FT4 and FT3 concentrations. Each surplus serum after clinical examination on the first, third, eighth and twenty-ninth day was stored at − 80 °C until analysis. The procedure of ultrafiltration LC-MS/MS is shown briefly below: 0.45 mL of serum was equilibrated for 5 min at 37 °C then ultrafiltrated by a centrifugal filter unit (Centrifree® Ultrafiltration Device, Merck, Germany) at 1300×*g* for 1 h at 37 °C. After ultrafiltration, 0.21 mL of the ultrafiltrate was spiked with T4-^13^C_6_ and T3-^13^C_6_ as internal standards, and then purified with a cartridge (Oasis® MCX, Waters corporation, USA). After the sample was evaporated to dryness, the residue was dissolved with HCOOH/40%methanol solution (1:100). The sample was subjected to a LC-MS/MS for determination of FT4 and FT3. The measurement ranges of FT4 and FT3 were 3.1–1225 pmol/L and 1.8–731.1 pmol/L, respectively.

Free thyroid hormone levels measured by ultrafiltration LC-MS/MS are also shown in Fig. 1 and Supplementary Table 1. The patient’s FT4 concentrations on the first, third, eighth and twenty-ninth day were 160.0 pmol/L, 135.5 pmol/L, 48.9 pmol/L and 0.4 pmol/L (below the limit of quantification), respectively. Her FT3 concentrations on each day were 17.8 pmol/L, 21.0 pmol/L, 13.9 pmol/L and 2.1 pmol/L, respectively. Whereas the level of FT3 was increased on the third day, the patient’s FT4 level was highest on the first day then decreased consistently over a period of 4 weeks.

## Discussion and conclusions

Levothyroxine is a commonly prescribed medication for hypothyroidism, and many cases of acute ingestion of excess levothyroxine have been reported, such as young children ingesting tablets by accident [3], adults attempting to commit suicide [4–7]. Edmundo K et al. described an adult case of accidental intoxication with 50 mg/day instead of 50 μg/day of levothyroxine over 9 days, due to pharmacist error in the preparation of the capsules. The patient presented with a stuporous mental state, atrial fibrillation and acute respiratory failure, but treatment with charcoal hemoperfusion was successful [8]. In most of the cases mentioned above, the patients was not in critical condition in spite of the excessive intake of levothyroxine, ranging from 2 mg to 720 mg. FT4 levels in those cases ranged from 38.7 pmol/L to > 167 pmol/L [3–7]. These reports suggested that clinical course severity did not always depend on the amount of levothyroxine ingested or on the patient’s free thyroid hormone level, though pre-hospital deaths were not included.

In terms of thyroid hormone action, more than 99% of T4 and T3 are pooled in serum in binding forms with thyroxine binding globulin, prealbumin and albumin [9]. Thyroid hormone activity is produced by FT3. Serum FT3 enters cytoplasm through thyroid hormone transporters, specific transporters for T3 or T4. Next, T3 is pooled in cytoplasm following conversion of T4 to T3 by iodothyronine deiodinase [10]. Finally, T3 binds to nuclear T3 receptors to initiate transactivation. Thus, there are various binding sites in serum and cytoplasm which are capable of storing T3 and T4. In the present case, the patient had a benign clinical course despite temporal toxic exposure to levothyroxine. As she had thyroid dyshormonogenesis, it was indicated that the prior discontinuation of levothyroxine for a month caused severe hypothyroidism before massive ingestion, which increased the potential vacant binding sites of thyroid hormone binding proteins as a buffer to prevent toxic T3 effect.

Ishihara T et al. reported an athyreotic patient having taken 2 mg of levothyroxine at one time, with serum concentrations of T4, FT4 and reverse T3 (rT3) that reached a peak on the second day, while the serum T3 level peaked 1 day later [7]. FT3 level in the present case also peaked later, indicating that the toxic dose of levothyroxine partly changed to T3 during the 2 days. Ishihara T et al. also pointed out that the maximum concentrations of T4, FT4 and rT3 were very high, while the peak T3 level did not exceed the upper limit of the normal range. No signs or symptoms of thyroid toxicity other than mild tachycardia were noted. The patient exhibited a low T3/T4 ratio, and it was speculated in that report that the thyroid type 1 deiodinase activity in the thyroid was one of the major determinants in the metabolic clearance of serum T4. Furthermore, there was a report that the mean FT3/FT4 ratios in Graves’ disease and destruction-induced thyrotoxicosis were 0.395 and 0.287, respectively [11]. Compared to that study, the FT3/FT4 ratios in the present case, 0.11 on the first day and 0.15 on the third day (Supplementary Table 1), were obviously low. Another group reported that the FT3/FT4 ratios after total thyroidectomy were lower than those before the surgery in patients with papillary thyroid carcinoma, demonstrating that the median ratio was 0.31 (0.28–0.34; 25th to 75th percentiles) on pre-thyroidectomy and 0.25 (0.22–0.28) on post-thyroidectomy [12]. It is known that almost 80% of T3 is derived peripherally from T4, and about 20% of T3 is secreted directly from the thyroid in healthy subjects. The half-life of T3 is about 1 day, which is much shorter than that of T4, which is about 7 days. Therefore, it is considered that T3 level in thyroid dyshormonogenesis could be increased remarkably only if there is a continuous, sufficient supply of T4. All T3 in our patient was considered to be derived from T4; thus, her FT3 level, which represented thyroid hormone activity, would had been lower than that of people with normal thyroid function in the same situation. Thus, it was reasonably speculated that the lack of intra-thyroidal T3 production caused by iodide organification defect could protect her against the overdose of levothyroxine.

Concerning methodology for measurement of thyroid hormone, various immunoassays are commercially available. Thirteen in vitro diagnostic manufacturers participated in the Phase IV methods comparison study of standardization of free thyroxine measurements, and the upper limits of measurement intervals for FT4 by those immunoassays were listed as 77–155 pmol/L [13]. Not only by levothyroxine overdose, but also in daily medical practice, we sometimes see a patient, typically one with Graves’ disease, whose FT4 or FT3 levels exceed the upper measurement limit. It is generally impossible by immunoassay to accurately determine real concentration when it is over the measurement range. Using LC-MS/MS, however, higher concentrations are able to be determined. As a result of measuring FT4 by ultrafiltration LC-MS/MS in the present case, it was revealed that FT4 level was tremendously increased compared to FT3 level on the first day, and was decreased on the third day. It should be noted that ultrafiltration is distinct from the standard method, which is equilibrium dialysis. Nevertheless, a previous comparison study evaluated an ultrafiltration plus LC-MS/MS assay for FT4, and reported that the correlation between the ultrafiltration LC-MS/MS and the gold standard equilibrium dialysis methods was excellent (*r* = 0.954) [14].

In the present case, the FT3 levels measured using ultrafiltration LC-MS/MS were differed from those using ECLIA. Currently, FT4 and FT3 measurements have not been standardized. The International Federation of Clinical Chemistry Committee for Standardization of Thyroid Function Tests compared the results for measurement of a panel of single donor sera using clinical laboratory procedures (17 for FT4, 14 for FT3) based on equilibrium dialysis-isotope dilution-mass spectrometry and immunoassays [15]. With regard to FT3, they reported that the biases were distributed from − 30 to + 22%. Therefore, free thyroid hormone levels would be disparate between LC-MS/MS and immunoassays including ECLIA.

While there is a consensus about the management of ordinary thyrotoxicosis, standards for treatment of patients with acute intake of excess levothyroxine have not been established. Therefore, it is possible that patients with a large amount of ingested levothyroxine may undergo unnecessary treatment. There were several reported cases whose FT4 levels were over the upper limit of measurement range of immunoassays, but underwent benign clinical course [3] [4] [5]. On the other hand, the previous reports described patients whose clinical condition were worsened later [3] [4] [5], suggesting that it is difficult to predict the future clinical course accurately only by symptoms and laboratory data immediately after the excessive ingestion of levothyroxine. Indeed, the FT3 level was also increased later in the present case. Lessons from these cases suggest that patients’ symptoms and condition may not totally depend on the amount of levothyroxine, and that the trend of both FT4 and FT3 level is helpful to understand the patient’s clinical course. It is essential that clinicians assess each patient’s condition appropriately not to provide overtreatment but rather adequate treatment. To that end, accurate and rapid measurement of extreme high level of free thyroid hormone, which is impossible by conventional immunoassays, is desirable. In addition, the previous report indicating that the signs and symptoms of thyrotoxicosis due to ingestion of excess liothyronine would appear more abruptly [16] suggests that the measurement of FT3 levels are more important. This report would be the first report clarifying the changes in extremely high level of FT4 and FT3 using ultrafiltration LC-MS/MS. Soldin OP et al. reported that LC-MS/MS had great potential to be applied in the routine clinical assessment of FT4 and FT3 in the near future [17]. When the measurement of high concentrations of free thyroid hormone by LC-MS/MS becomes common in the future, more beneficial data for the management of excessive ingestion of thyroid hormones can be obtained.

In conclusion, the serum level of FT4 after the ingestion of 5 mg of levothyroxine in the present case with hypothyroidism increased above the measurable range of immunoassay. More specific FT4 concentrations were determined using ultrafiltration LC-MS/MS, which revealed that the value of FT4 was extremely high compared to that of FT3. These results indicate that ultrafiltration LC-MS/MS is worth utilizing for measuring free thyroid hormone levels in thyrotoxic cases when the level is over immunoassay’s upper measurement limit. Though LC-MS/MS is currently a time-consuming technique, it is expected to be developed into a routine method in clinical laboratories in the near future, which will lead to more appropriate management of extreme thyrotoxicosis.

## Supplementary information

**Additional file 1: Table S1.** Dose of levothyroxine and the values of TSH, FT4 and FT3.

## Data Availability

All data generated or analysed during this study are included in this published article.

## Abbreviations

LC-MS/MS: Liquid chromatography-tandem mass spectrometry
(F)T4: (free) thyroxine
(F)T3: (free) triiodothyronine
TSH: Thyroid stimulating hormone
ECLIA: Electrochemiluminescence immunoassay
rT3: Reverse triiodothyronine
