# Supplementary material for: Time-dependent changes in FT4 and FT3 levels measured using mass spectrometry after an acute ingestion of excess levothyroxine in a case with hypothyroidism
Source: Thyroid Res. 2020 May 1;13:4. doi: 10.1186/s13044-020-00078-7 (PMC7193347; doi:10.1186/s13044-020-00078-7)
Supplement: Supplementary file 1 — Additional file 1: Table S1. Dose of levothyroxine and the values of TSH, FT4 and FT3. [file 13044_2020_78_MOESM1_ESM.docx]

Supplementary Table 1. Dose of levothyroxine and the values of TSH, FT4 and FT3.

|  | | One year before  the visit | One month before  the visit | Two days before  the visit | One day before  the visit | 1^st^ day | 3^rd^ day | 8^th^ day | 29^th^ day |
| --- | --- | --- | --- | --- | --- | --- | --- | --- | --- |
| Levothyroxine (µg/day) | | 150 | 0 | 2000 | 2000 | 1000 | 0 | 0 | 0 |
| ECLIA | |  |  |  |  |  |  |  |  |
| TSH (mIU/L) | ref. 0.500-5.000 | 0.156 |  |  |  | 2.620 | 0.207 | 0.073 | 66.500 |
| FT4 (pmol/L) | ref. 12-22 | 23.4 |  |  |  | > 100 | > 100 | 48.9 | 2.6 |
| FT3 (pmol/L) | ref. 3.5-6.1 | 5.1 |  |  |  | 24.5 | 28.8 | 16.4 | 2.4 |
| Ultrafiltration LC-MS/MS | |  |  |  |  |  |  |  |  |
| FT4 (pmol/L) |  |  |  |  |  | 160.0 | 135.5 | 48.9 | (0.4) ^#1^ |
| FT3 (pmol/L) |  |  |  |  |  | 17.8 | 21.0 | 13.9 | 2.1 |
| FT3 / FT4 |  |  |  |  |  | 0.11 | 0.15 | 0.28 |  |

#1: Under the limit of quantification of FT4
